# Surface Properties of *Helicobacter pylori* Urease Complex Are Essential for Persistence

**DOI:** 10.1371/journal.pone.0015042

**Published:** 2010-11-29

**Authors:** Tobias D. Schoep, Alma Fulurija, Fayth Good, Wei Lu, Robyn P. Himbeck, Carola Schwan, Sung Sook Choi, Douglas E. Berg, Peer R. E. Mittl, Mohammed Benghezal, Barry J. Marshall

**Affiliations:** 1 Ondek Pty Ltd and H. pylori Research Laboratory, Microbiology and Immunology, University of Western Australia, Nedlands, Australia; 2 Biochemisches Institut, Universität Zürich, Zürich, Switzerland; 3 Department of Molecular Microbiology, Washington University School of Medicine, St. Louis, Missouri, United States of America; University of Hyderabad, India

## Abstract

The enzymatic activity of *Helicobacter pylori's* urease neutralises stomach acidity, thereby promoting infection by this pathogen. Urease protein has also been found to interact with host cells *in vitro*, although this property*'*s possible functional importance has not been studied *in vivo*. To test for a role of the urease surface in the host/pathogen interaction, surface exposed loops that display high thermal mobility were targeted for inframe insertion mutagenesis. *H. pylori* expressing urease with insertions at four of eight sites tested retained urease activity, which in three cases was at least as stable as was wild-type urease at pH 3. Bacteria expressing one of these four mutant ureases, however, failed to colonise mice for even two weeks, and a second had reduced bacterial titres after longer term (3 to 6 months) colonisation. These results indicate that a discrete surface of the urease complex is important for *H. pylori* persistence during gastric colonisation. We propose that this surface interacts directly with host components important for the host-pathogen interaction, immune modulation or other actions that underlie *H. pylori* persistence in its special gastric mucosal niche.

## Introduction

*H. pylori* chronically infects the gastric mucosa of billions of people worldwide, causes peptic ulcer disease in 10% or more of them, and is also implicated as an early critical risk factor for gastric cancer, one of the most frequently lethal malignancies in human populations [1]. One of the first characterised factors essential for colonisation by *H. pylori* was urease, an abundant enzyme that decreases the acidity of *H. pylori'*s immediate environment by generating ammonia and carbonate from the urea we secrete as metabolic waste [2], [3]. Although such local control of gastric acidity is considered essential, urease-negative *H. pylori* strains were unable to colonise piglets whose acid secretion had been suppressed, suggesting an additional role for urease [4]. Possible explanations include use of ammonia that urease generates to synthesise essential metabolites, especially amino acids [5]; protection from peroxynitrite [6], enhanced survival in macrophages [7]; evasion of phagocytosis [8] and complement mediated opsonisation [9]. Quite a different explanation invokes urease-host tissue interactions, independent of enzymatic activity, and is based on *in vitro* studies that detected urease activation of macrophages [10], monocytes [11], blood platelets [11], dysregulation of gastric epithelial tight junctions [12] and induction of cytokine production from gastric epithelial cells [13] through binding to CD74 (MHC class II invariant chain) [14]. *H. pylori* urease consists of a dodecamer of UreA-UreB subunits (26.5 and 61.7 kDa, respectively), assembled as four alpha/beta trimers, producing a ball-like supramolecular structure [15], [16]. We propose that properties of the dodecamer surface contribute to urease*'*s acid stability [15] and host interactions. We tested the role of the urease surface in *H. pylori*/host interactions, and found that surface regions of this enzyme in which changes that did not affect enzymatic activity impaired bacterial persistence in a murine experimental infection model.

## Results

### Urease Altered on the Surface can Retain De-acidification Function

**Figure 1:**
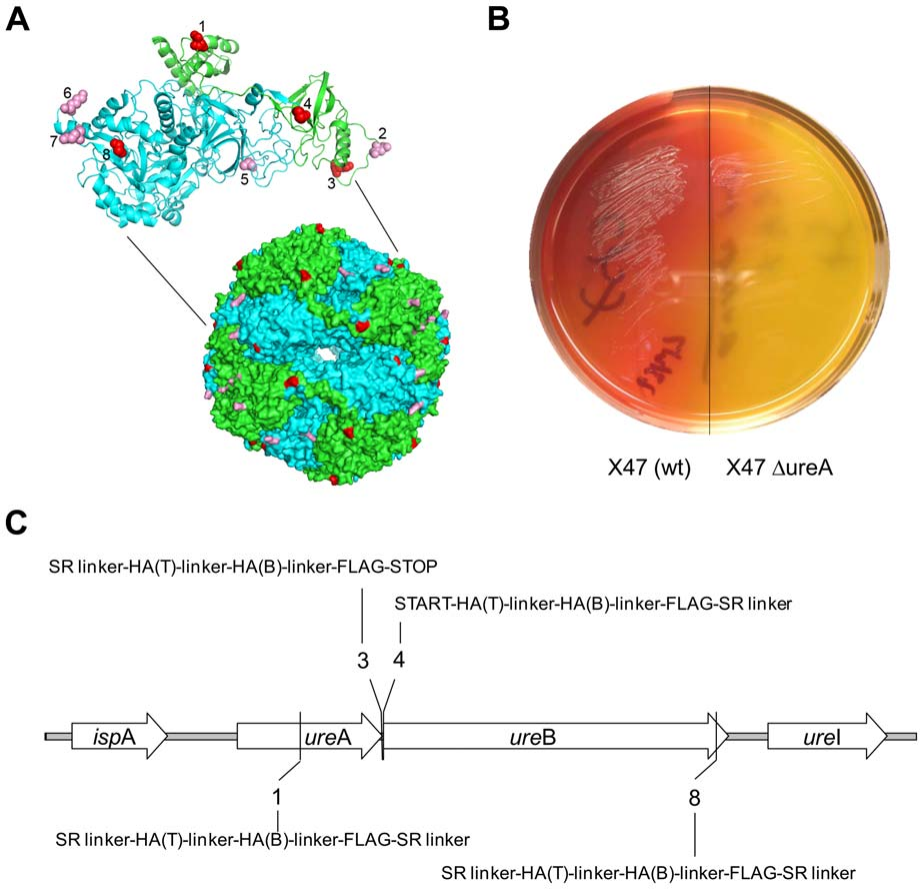
Recombinant regions of urease and selection for enzyme function. **a**) Molecular structure of urease showing insertion sites on the surface of urease. Urease subunit A (green) and subunit B (blue) associate to form a dodecameric supramolecular molecule [15], [16]. Sites 1 to 8 correspond to residues 102, 231 and 238 from UreA and residues 1, 66, 326, 541 and 549 from UreB, respectively. Insertion sites 1, 3, 4, and 8 are indicated in red. Urease activity could not be retained when altered at sites 2, 5, 6, and 7 (pink). **b**) Selection of bacteria producing functional urease on acidified media supplemented with the urease substrate, urea. Left side: X47 wild type; the colour change observed on the left side indicated that bacterial colonies were producing functional urease and growing. Right side: X47 Δ*ureA*: there was no colour (X47 wild-type). Colour change did not occur on the right side, indicating that inoculated colonies were unable to grow or functional urease was not being produced (X47 Δ*ureA*). **c**) A schematic showing insertion sites at the urease locus of DNA coding epitopes and linker***s.*** Insertions were made in DNA corresponding to insertion after amino positions 102 (site 1) and 238 (site 3) of UreA (GenBank AAD07144.1), and amino acid positions 1 (site 4) and 549 (site 8) of UreB (GenBank AAD07143.1). Insertions at sites 3 and 4 correspond to the C- and N-termini of UreA and UreB, respectively. DNA coded HA(T): hemagglutinin T cell eptitope; HA(B) hemagglutinin B cell epitope; SR linker: semi-random linker; linker: GPSL linker; FLAG: FLAG epitope; STOP: STOP codon.

To test the possible involvement of the urease surface in host-pathogen interaction, we generated *H. pylori* with inframe insertions at eight sites in urease. First the UreA/UreB structure [15] was analysed *in silico* to identify surface regions that might tolerate the insertion of two epitope tags (Figure 1a). Mutant *H. pylori* with in frame insertions of DNA encoding epitope tag sequences at eight specific sites in chromosomal *ureA* and *ureB* genes were then generated by a PCR and transformation method. The sites chosen were those corresponding to the N and C-termini of UreA and UreB, respectively and six additional regions in which structural considerations suggested that modest sequence changes would not necessarily inactivate urease*'*s enzymatic activity. To reduce structural stresses resulting from epitope tag insertion the tags were separated from retained urease sequences by a flexible amino acid linker. The tagged region was also flanked by semi-random six amino acid linkers whose underlying DNAs had been designed to exclude two of the three translation termination codons.

**Figure 2:**
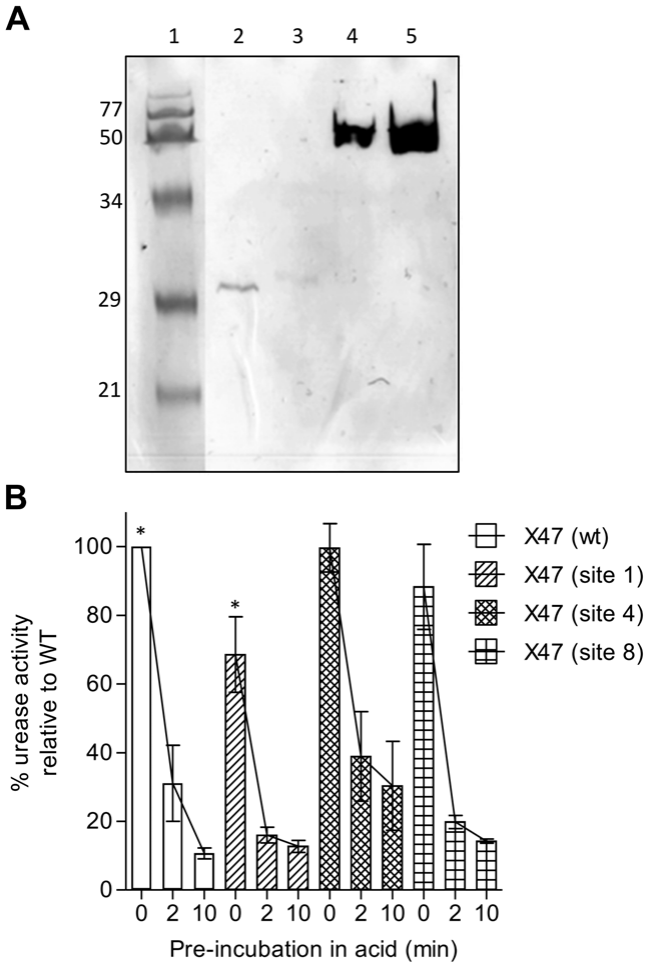
Recombinant urease activity and acid stability. **a**) Western Blot analysis of *H. pylori* producing urease with insertions at sites 1, 3, 4 or 8. Lanes 1: Maker (MW: KDa shown); Lane 2: X47 (site 1); Lane 3: X47 (site 3); Lane 4: X47 (site 4); Lane 5: X47 (site 8). Mutant urease was detected using anti-FLAG antibody, directed against UreA (lanes 2 and 3) or UreB (lanes 4 and 5) mutants. **b**) Ability of permeabilised bacteria expressing wild-type or mutant urease to neutralise acid after incubation at pH 3 in the presence of urea. To determine acid stability and activity of wild-type and mutant ureases, bacteria were incubated at pH 3 for 0, 2 or 10 min prior to assay of urease activity. After pre-incubation the solution pH was adjusted to pH 7, neutralised, urea was added as substrate and urease activity was measured by a change in pH, as indicated by a change in the colour of phenol red. Significantly reduced urease activities independent of pre-incubation at pH 3 are annotated “ * ” (Student*'*s T-test, 2 tailed, equal variance). SEM displayed (n = 3).

Four of eight candidate sites yielded mutant urease enzymes that allowed *H. pylori* to hydrolyse urea (Figure 1b; Figure 1c). Insertions at the other four sites did not result in isolation of bacteria expressing functional ureases under these conditions. Western blot analysis confirmed that *H. pylori* producing mutant urease at sites 1, 3, 4 or 8 contained insertions of epitope tags (Figure 2a). The pH of the mouse stomach lumen, which *H. pylori* must traverse to establish gastric mucosal infection, is between 3 and 4 [17]. To determine if these insertions in urease*'*s surface exposed loops altered its activity or stability we assayed enzymatic activity in bacteria expressing wild-type or mutant urease after exposure to acid (pH 3). One of the mutant ureases (insertion at site 1) was more sensitive than wild type, and the three other mutant ureases were similar to wild type in their sensitivity to this acid treatment (Figure 2b; Student*'*s T-test; p<0.05).

### Altering Surface Properties of Urease Alters Persistence in the Host

**Figure 3:**
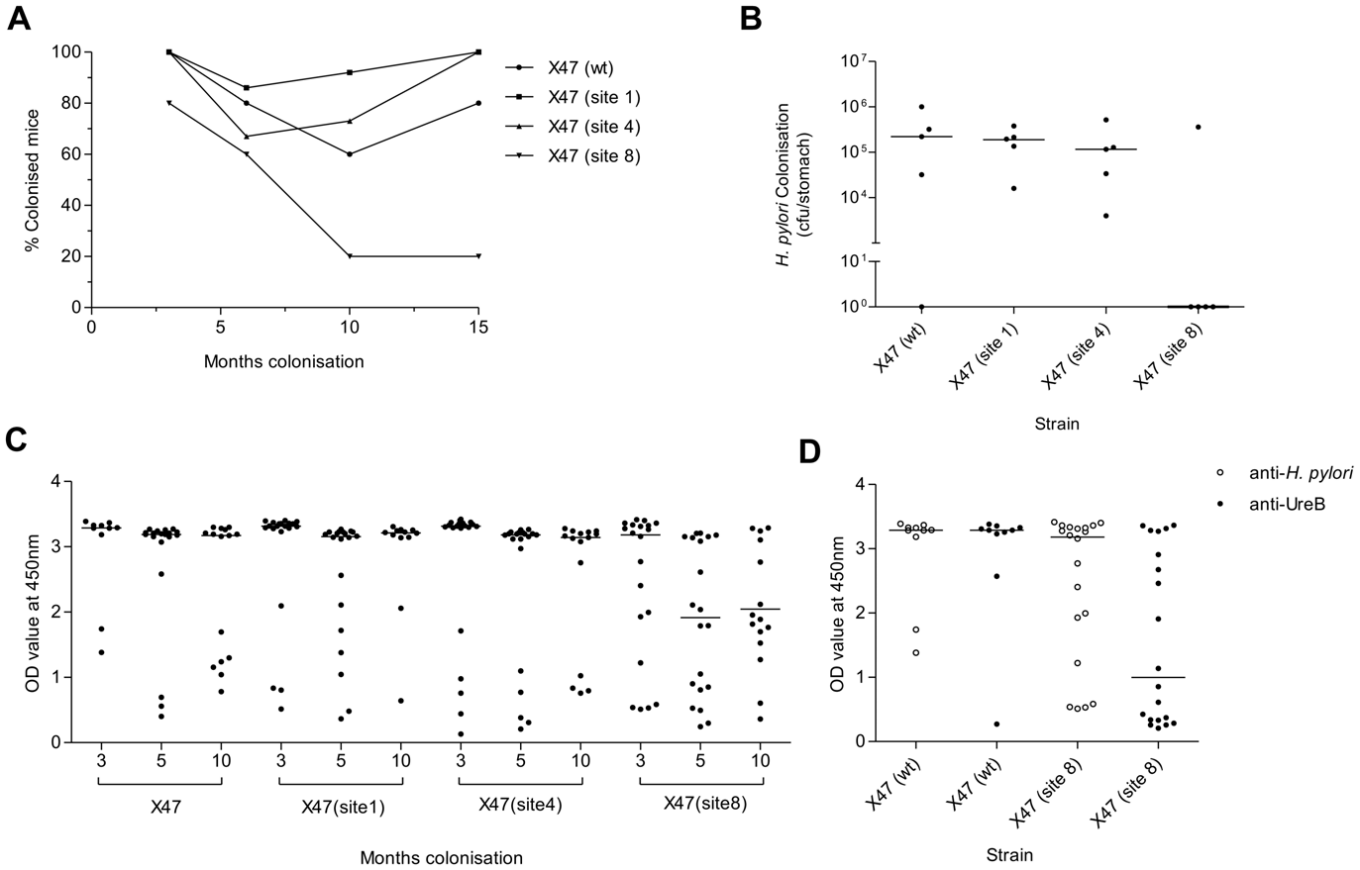
Insertion mutant but active urease can affect bacterial colonisation. **a**) Persistence over 15 months expressed as percentage of colonised mice at each time point (n = 5–15). **b**) Colonisation level of mice infected with *H. pylori* expressing mutant urease after 15 months (n = 5; median displayed). **c**) Persistence of *H. pylori* expressing mutant urease as indicated by anti-*H. pylori* IgG levels (n = 12–20; median displayed). Strains were recombinant at either sites 1, 4 or 8 in urease. **d**) Comparison of anti-*H. pylori* IgG and anti-UreB IgG levels resulting from colonisation of mice for 3 months with X47 expressing wild-type urease, X47 (wt), or urease mutant at site 8, X47 (site8), (n = 10–20; median displayed).

**Figure 4:**
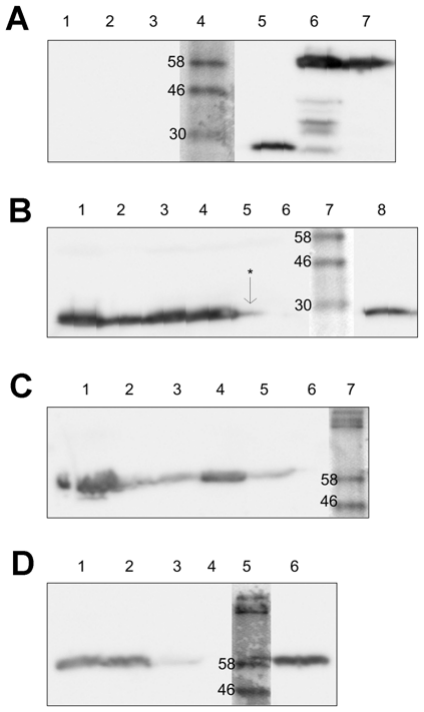
Insertion mutant ureases are stable during persistent infection. Western Blot analysis of *H. pylori* cultured from mice after 10 months colonisation, probed with anti-FLAG antibody. Where described, each lane represents protein extracted from a pool of bacteria harvested from an individual mouse. **a**) lanes 1–3: pools of X47 (wt) from individual mouse; lane 4: molecular size marker (MW: KDa shown); lane 5: X47 (site 1); lane 6: X47 (site 4); lane 7: X47 (site 8). **b**) lanes 1–5: pools of X47 (site 1) from individual mouse; lane 7: molecular size marker; lane 8: X47 (site 1). **c**) lanes 1–5: pools of X47 (site 4) from individual mouse; lane 7: molecular size marker. **d**) lanes 1–3: pools of X47 (site 8) from individual mouse; lane 5: molecular size marker; lane 6: X47 (site 8). To confirm protein sample integrity urease activities were assayed in wild-type X47 and in a pool of X47 insertion (site 1 mutant) from mice in which FLAG expression was not detected (annotated *; data not shown). Molecular of standard proteins (KDa) are shown directly adjacent to the marker.

The ability of each mutant urease-producing *H. pylori* strain to colonise C57BL/6 mice was tested. The strain with site 3-mutant urease did not colonise mice (data not shown) and was not studied further, whereas each of the other three mutant strains colonised mice as efficiently as the wild-type in short-term (<3 months) infections. However, persistence of *H. pylori* with site 8-mutant urease was greatly reduced over longer periods of time (Figure 3a). In confirmation, bacterial titres in mouse stomachs and anti-*H. pylori* IgG in serum were each much reduced relative to wild type and the other two mutants in the case of ten month infection by this strain (Figure 3b, c). We note that *H. pylori* expressing urease containing the recombinant at site 8 insertion, which was present in infected animals at 3 months of infection at bacterial load similar to that of bacteria expressing wild type urease (Figure 3a), exhibited a weaker humoral response against the urease B subunit (UreB) than did the wild type strain, but a normal response against total *H. pylori* antigen (Figure 3d). Important in our infection protocol, each inoculation used a pool of three independent transformant clones. This rules out concerns of possible bacterial attenuation by secondary chromosomal mutations distinct from the insertions within urease. Further tests showed that nearly all *H. pylori* recovered from mice 10 months after inoculation still expressed the expected mutant urease, which showed that this persistent colonisation was not due to loss of inserted DNAs. As the sole exception, just one mouse initially infected with the site 1 mutant strain seemed to produce revertant urease at ten months (Figure 4). It was also striking that reduced urease activity measured *in vitro* did not correlate well with reduced colonisation ability. For example, the site 1 mutant strain, whose urease was less acid stable than the others, colonised mice as well as wild-type, and the site 8 mutant, whose urease activity was similar in acid resistance to that of wild type, was nevertheless less persistent in mouse colonisation. Rather, we propose an alternative role for the urease surface around site 8 (Figure 1a), important during chronic infection.

## Discussion

In summary, a structure-based insertion mutagenesis of the urease complex identified two discrete regions on the enzyme surface that are needed for colonisation or persistence of *H. pylori*, site 3 and site 8 (Figure 1a). The inability of mutant *H. pylori* to colonise mice due to an insertion at site 3 gives further support for an alternative role of urease, different from acid neutralisation and warrants further investigation. In addition, our results suggest that the site 8 region is not essential for the de-acidification function of urease, but rather is involved in an alternative function required for persistence of infection in the host. Site 8 is located in the turn of a beta-meander of the urease complex surface. No insertions or deletions are found at this site in homologous urease sequences in diverse organisms (Figure S1) and we therefore propose that the precise structure of this beta-meander is important for urease*'*s alternative role during long-term colonisation. Sites 3 and -8 have in common the feature that both are located in proximity to the rotation axis that connects three alpha/beta trimers. Since enzymatic activity relies on the integrity of the alpha/beta trimer, the insertion of tags at sites 3 and -8 could modify the trimer/trimer interaction surface without compromising the enzymatic activity.

Adaptive immune responses change dramatically during the establishment and maintenance of chronic *H. pylori* infection, in particular at the site of infection. Urease site 8 overlaps with an established *H. pylori* CD4^+^ T-cell epitope, in response to which splenic lymphocytes produce cytokine IL-4 [18], a promoter of Th_2_ responses and driver of antibody production. In contrast the sequences at sites 1, 3 and 4 do not coincide with any known B or T cell epitopes. Since urease constitutes up to 10% of bacterial protein [2], [3] the removal of an abundant Th_2_ driver may prevent adequate immune modulation by *H. pylori* important for persistence and thereby facilitate bacterial clearance by further increasing the Th_1_ bias during *H. pylori* infection. The observations that *H. pylori* interferes with dendritic cells [19] and that CD74 plays a role in regulation of the motility of dendritic cells [20] are in line with a role of urease in the modulation of the immune response to achieve persistence. This hypothesis is further supported by the observation that endogenous CD74 receptor ligands or ligands from human pathogens function as trimers [21], [22] and an inference that insertions of tags at sites 3 and -8 could affect the trimerisation of urease alpha/beta trimers. Alternatively, given that binding of *H. pylori* urease to CD74 on the gastric epithelium increases IL-8 secretion and up regulation of inflammatory cytokines [23] we can imagine that modification of the urease site 8 region interferes with a direct interaction with CD74-type host cell receptors, and reduces the pro-inflammatory response (which contributes to tissue damage, and thereby release of metabolites on which *H. pylori* is thought to feed) and thereby prevents chronic infection. The modification of the site 8 epitope in the recombinant urease also might interfere with urease*'*s chemotactic activity abrogating the inflammatory cell response. This scenario is compatible with the penetration of urease into the lamina propria where it is in close proximity to phagocytic cells [24] and with the marked mucosal infiltration by polymorphonuclear leukocytes, macrophages and lymphocytes during *H. pylori* infection and resultant characteristic persistent gastritis. Further studies examining the role of the urease surface in these interactions are required to identify precisely what is the target receptor of the urease surface at site 8 region and to elucidate the mechanism underlying the urease surface-mediated *H. pylori* persistence.

### Conclusions

**Figure 5:**
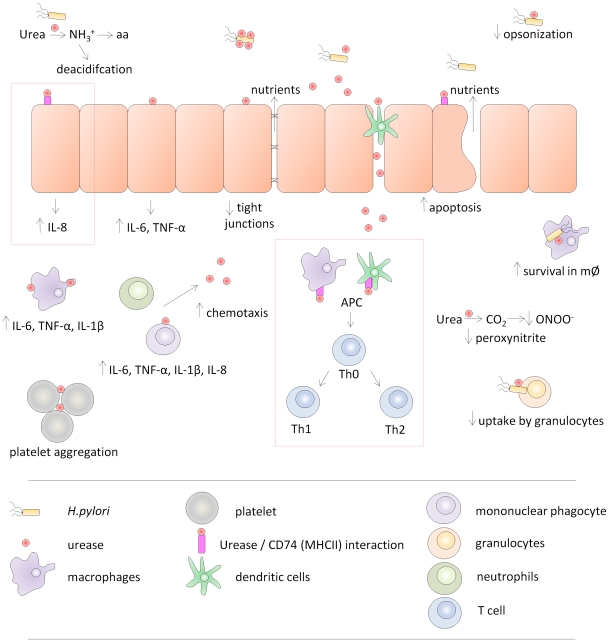
Proposed multiple roles of Helicobacter pylori urease during infection. To explain the loss of ability to persist in strains containing an insertion at urease surface site 8, we suggest a model of immune modulation mediated by the urease interaction with host tissue CD74 receptor. This could lead to a reduction of the pro-inflammatory response elicited by the epithelial cells or to an increase in the Th_1_ bias via the dendritic cells (red boxes). Other non-enzymatic functions of urease that might contribute to persistence include facilitation of resistance to complement mediated opsonisation [9], decreased uptake of *H. pylori* cells by granulocytes [8], increased *H. pylori* survival in macrophages [6], [7], and increased release of nutrients via compromised tight junctions [12] and/or apoptosis of epithelial cells [32].

In conclusion, the urease complex is multifunctional. The surface properties of this protein, distinct from the ureolytic activity *per se*, were found to be important for *H. pylori* colonisation and persistence. We hypothesise that the modification of the urease surface at site 8 compromises CD74-mediated immune modulation, reduces the pro-inflammatory response elicited by the epithelial cells or increases the Th_1_ bias via the dendritic cells. Alternatively, other receptors, or yet unidentified mechanisms, might be involved in the urease surface-mediated *H. pylori* persistence. Figure 5 presents one appealing view of the multiple roles attributed to the urease complex, including urea hydrolysis, opsonisation, platelet aggregation, survival in macrophages, chemotaxis and immune modulation through CD74. Given the many failed efforts to develop anti-*H. pylori* vaccines to date, a better molecular understanding of factors important in persistence should contribute to development of new, much needed therapeutic approaches.

## Materials and Methods

### Bacterial Strains and Growth Conditions

Streptomycin resistant *H. pylori* strain X47 was used for all experiments [25]. Bacteria were grown on Brain Heart Infusion (BHI) based agar plates supplemented with 5% horse blood and when appropriate, with erythromycin (10 µg/mL) or streptomycin (10 µg/mL) in an atmosphere containing 5% CO_2_. *H. pylori* producing functional urease were selected on BHI based agar plates supplemented with 7% (v/v) horse serum. Phenol, red (100 mg/L), and urea (600 mg/L) were added, and the molten agar was acidified to pH 5 using 1M HCl when needed to select for *H. pylori* producing functional urease, as illustrated in Fig. 2. DNA containing the *rpsL,ermB* (selectable and contraselectable cassettes [26]) flanked by segments of urease gene DNA (for homologous recombination), at the urease locus, were produced using splicing by overlap extension PCR [27] and inserted at the desired sites in chromosomal *ureA or ureB* genes of a *ΔureAB-rpsL.erm* derivative of *H. pylori* strain X47 [25] using natural transformation and homologous recombination. DNA encoding HA epitopes (aa 150–159 and 110–120 of Influenza virus A/PR/8/34 hemagglutinin protein [28]), flexible linkers (described below) and a FLAG tag were similarly fused to regions of DNA for homologous recombination and transferred to modified *H. pylori*, replacing the *rpsL.ermB* counter selection cassette.

### Structural Biology

In order to predict sites in urease which were tolerant to epitope insertion, the three-dimensional structure of the *H. pylori* urease complex was reconstructed from deposited coordinates (PDB-id: 1e9y) by applying the crystallographic symmetry operators. The urease complex, consisting of 12 UreA and 12 UreB polypeptide chains, was visually inspected for surface exposed loops showing high thermal mobility likely to tolerate insertions of epitope tags. The average temperature factors for main chain atoms of sites 1 to 8 where 64.3 Å^2^, 70.0 Å^2^, 36.6 Å^2^, 52.9 Å^2^, 23.3 Å^2^, 105.0 Å^2^, 59.6 Å^2^, and 61.8 Å^2^ whereas the average temperature factors for main chain atoms of UreA and UreB where 43.8 Å^2^ and 34.9 Å^2^, respectively.

### Splicing by Overlap Extension (SOE) PCR

All PCR constructions used 26695 genomic DNA as template for initial amplifications. Primers used in this study are shown in Table S1 and the combinations used are shown in Table S2. The principles of splicing by overlap PCR have been previously reported [27]. To construct *rpsL,ermB* cassette [29] flanked by regions for homologous recombination 3 stages of PCR were performed. Stage 1 involved the amplification of the *rpsL,ermB* cassette, and flanking regions for homologous recombination at the urease locus. Stage 2 involved the addition of either flanking region to the *rpsL,ermB* cassette using 2 way SOE PCR, Stage 3 involved using these products as template to add both flanking regions to the *rpsL,ermB* cassette using 2 way SOE PCR (Table S2)**.** To produce DNA encoding HA (aa 150–159 and aa 110–120 of Influenza virus A/PR/8/34 hemagglutinin protein; [28]) and FLAG (DYKDDDDK) epitopes separated by a four amino acid linker, flanked by semi-random six amino acid linkers and regions for homologous recombination, 3 stages of PCR were performed using AccuPrime™ Pfx Supermix (Invitrogen). Stage 1 involved the addition of flanking linkers, stage 2 involved the addition of HA and FLAG epitopes, stage 3 involved the addition of flanking regions for homologous recombination using 2-way SOE PCR (Table S2). Thermocycling conditions were as follows: 94°C for 15 s, 56°C for 20 s, 68°C for 3.5 min (10 cycles). After the addition of primers an additional 35 cycles of 94°C for 15 s, 62°C for 20 s, 68°C for 3.5 min were performed, followed by a final extension of 3 min. Extension times were varied according to PCR product lengths.

### Natural Transformation of *H. pylori*

Overnight cultures of *H. pylori* X47 *ΔureAB rpsL.erm* grown on BHI based agar plates were subcultured onto plates supplemented with DENT (Oxoid) in lawns of approximately 2 cm in diameter. PCR products were *Dpn*I treated to remove residual genomic DNA and purified QIAQuick PCR Purification Kit (Quiagen) prior to use in transformation. Transformation was performed by the addition of approximately 1 µg of purified PCR product after growth of bacterial lawns for 6–8 hrs. After overnight incubation putative transformants were streaked on selective media. Bacteria harbouring functional urease containing permissive linkers were selected from pools of transformant bacteria by streaking on acidified media supplemented with the urease substrate, urea.

### Experimental Infection of Mice

C57BL/6, *Helicobacter* free, mice were purchased from the Animal Resource Centre (Perth, Western Australia). Studies were performed with approval from the UWA Animal Ethic Committee (approval no. 07/100/598). Eight week old mice were orogastrically inoculated with approximately 1.0×10^9^ *H. pylori* harvested from an overnight agar plate based culture into BHI broth (Oxoid). Mice were inoculated with pools of 3 independent, genetically characterised clones expressing wild type or mutant urease. To determine the level of colonisation, stomachs were harvested from sacrificed animals and opened, and residual food was removed. Opened stomachs were suspended in 500 µL PBS and homogenised using 5 mm stainless steel beads for 25 seconds at setting of 30 (Qiagen Tissue Lyser). Samples were then homogenised for a further 2 min at a setting of 10 to facilitate bacterial release from the tissue. Serial dilutions of homogenates were plated on BHI based agar plates supplemented amphotericin B (8 µg/mL), trimethoprim (5 µg/mL) and vancomycin (6 µg/mL), nalidixic acid (10 µg/mL), polymyxin B (10 µg/mL) and bacitracin (200 µg/mL) (24) and incubated under microaerobic conditions. Bacterial growth was scored 5–7 days later.

### SDS-PAGE and Western Blot Analysis

*H. pylori* were harvested from mouse stomachs, grown for 4 d, harvested and resuspended in SDS-PAGE loading buffer. Standard SDS-PAGE and Western Blot methodologies were performed [30]. Electrophoresis was performed using SDS-PAGE on a discontinuous 10% gel. For Western Blotting, proteins were transferred to PVDF Immuno-Blot PVDF (0.2 µM) membrane (Biorad). Membranes were blocked overnight at 2% Blocking Reagent (Roche) in Maleic acid buffer (100 mM Maleic acid, 150 mM NaCl, pH 7.5, 20°C) supplemented with 0.2% (v/v) Tween 20. To detect FLAG membranes were probed with a 1∶1000 dilution of monoclonal anti-FLAG (Sigma Aldrich) in 1% Blocking Reagent supplemented with 0.1% (v/v) Tween 20 for 2 hours at room temperature. For detection, membranes were incubated with rabbit anti-mouse IgG conjugated to horse radish peroxidase (Jackson ImmunoResearch Laboratories, Inc.) under identical conditions for 1 h at room temperature. Detection was performed using Chemiluminescent Peroxidase Substrate-3 (Sigma-Aldrich) and the FujiFilm LAS-3000 Imager. Urease was similarly probed using a 1∶200 dilution anti-urease alpha subunit (bc-14; Santa Cruz Biotechnology) and detected using a 1∶2500 dilution of rabbit anti-goat HRP conjugate antibody (Jackson ImmunoResearch Laboratories, Inc.).

### Enzyme-Linked ImmunoSorbent Assay (ELISA)

Mice sera were collected at different time points and assessed for the presence of urease specific IgG. Nunc 96 well maxisorb plates were coated with 10 ug/mL of Urease B protein (expressed from plasmid pILL927 and purified as described in reference [31]) in 100 µL carbonate buffer and incubated overnight at 4°C. Plates were washed 5 x with PBS supplemented with 0.05% (v/v) Tween 20 (PBST) and then blocked with 200 uL of PBS supplemented with 2% BSA (w/v) for 2 hours at 37°C. Following 2 x washes with PBST a 1∶20 dilution mouse sera in 100 uL of PBST supplemented with 2% (w/v) BSA was added to duplicate wells and the plates incubated for 1 hour at room temperature. Subsequently, plates were washed 5x with PBST and then a 1∶1000 dilution of anti-mouse IgG alkaline phosphatase (Sigma Aldrich) in 100 uL PBST supplemented with 2% (w/v) was added to each well and the plates incubated at room temperature for 1 hour. After 5x washes with PBST, 200 uL of nitrophenyl phosphate substrate in diethanolamine buffer was added to each well and the plates incubated for 40 min at room temperature in the dark before absorbance was measured at 405 nm.

### Urease Stability and Activity

*H. pylori* were harvested after growth for 24 h on BHI base agar plates and were rinsed in cold saline (0.9% v/v). The bacterial suspension was diluted to an OD_600_ of 4 and 15 µL was added to 15 µL of saline supplemented with Tween 20 (0.2% v/v). To each sample 90 µL of KCl (200 mM; pH 3) was added and samples were incubated for 10 min while shaking at 300 rpm at room temperature. Subsequently the solution was neutrali*s*ed by the addition of 120 µL of PBS (pH 6.8). 150 µL of each sample was added to 25 µL of phenol red sodium salt (80 mg/mL) and warmed to 37°C. The reaction was initiated by the addition of 75 µL of 0.5 M urea and the change in pH was measured by reading absorbance at 560 nm every 70 s.

## Supporting Information

Figure S1 **Alignment showing conservation of ureases at region of site 8.** Alignment of *H. pylori* UreB (sp|P69996) at the region of site 8, for which the crystal structure has been determined (PDBe Entry: 1e9y), and ureases from different species. UniProtKB/Swiss-Prot numbers are displayed.
(TIF)

Table S1 **Sequences of oligonucleotides used in this study.**
(DOC)

Table S2 **Primer combinations used to produce modified DNA.** Primers amplified either segments of regions coding exogenous DNA (to be inserted into urease genes) or regions used for homologous recombination after transformation (left flank and right flank).
(DOC)
