# Supplementary figures and images for: Surface Properties of Helicobacter pylori Urease Complex Are Essential for Persistence
Source: PLoS One. 2010 Nov 29;5(11):e15042. doi: 10.1371/journal.pone.0015042 (PMC2993952; doi:10.1371/journal.pone.0015042)

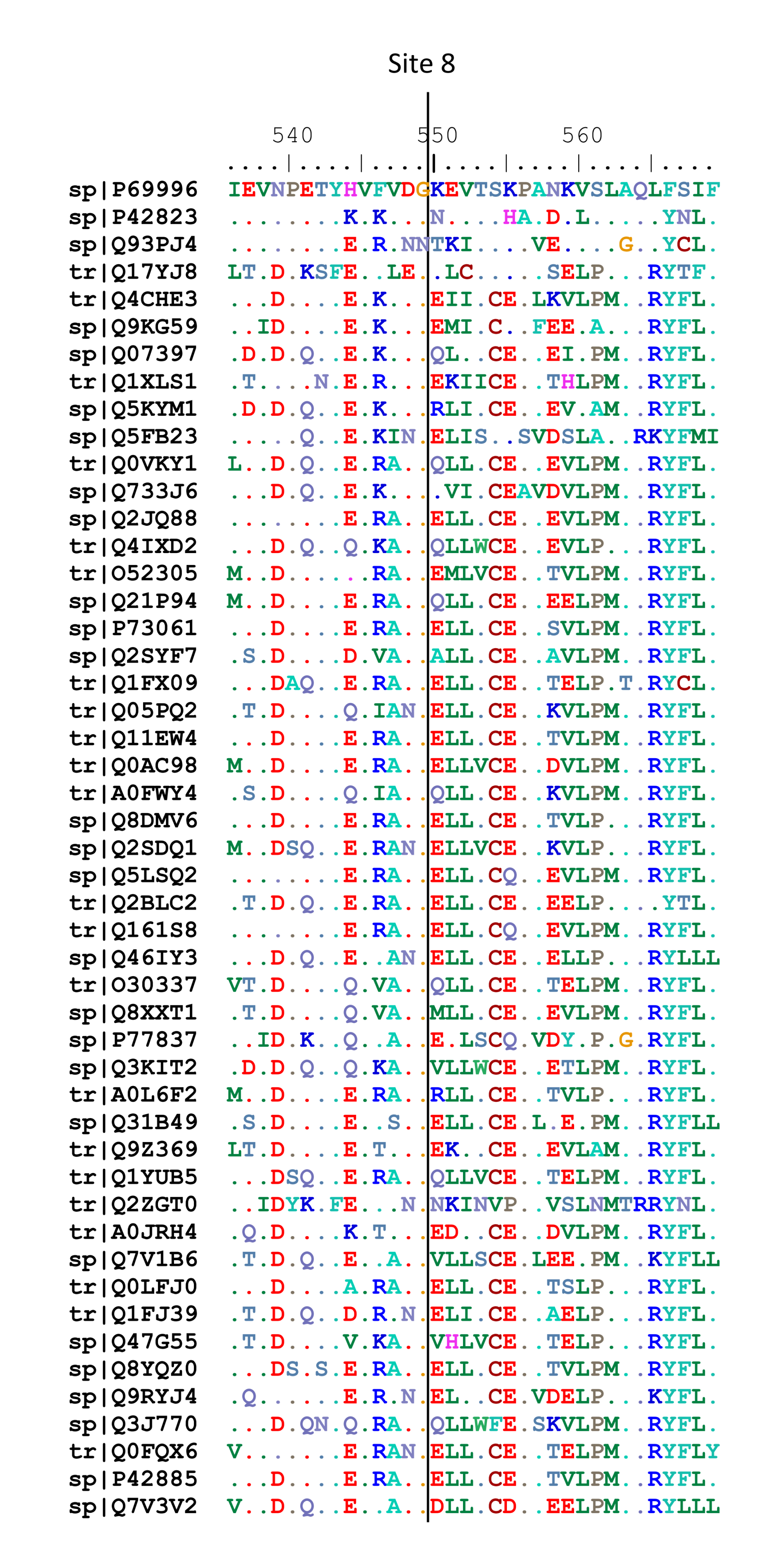

Supplement: Figure S1 — Alignment showing conservation of ureases at region of site 8. Alignment of H. pylori UreB (sp|P69996) at the region of site 8, for which the crystal structure has been determined (PDBe Entry: 1e9y), and ureases from different species. UniProtKB/Swiss-Prot numbers are displayed. (TIF) [file pone.0015042.s001.tif]
