# Supplementary material for: Surface Properties of Helicobacter pylori Urease Complex Are Essential for Persistence
Source: PLoS One. 2010 Nov 29;5(11):e15042. doi: 10.1371/journal.pone.0015042 (PMC2993952; doi:10.1371/journal.pone.0015042)
Supplement: Table S1 — Sequences of oligonucleotides used in this study. (DOC) [file pone.0015042.s002.doc]

**Table S1.** ***Sequences of oligonucleotides used in this study.***

| Primer | Sequence (5’ – 3’) |
| --- | --- |
| UF5-234 | gtgtttaatccatagttataaagcatcccattggcctcaataggggtat |
| UR2-234 | ttcaatagctataaattatttaataagtaacggtggcggtaaaaccct |
| UR2-56 | ttcaatagctataaattatttaataagtaagtgagcttggcgcaactcttta |
| UF5-56 | gtgtttaatccatagttataaagcatcgtgagcggtagtgtcgttgaat |
| *rpsL*-F | gatgctttataactatggattaaacac |
| ermR | Ttacttattaaataatttatagctattgaattacttattaaataatttatagctattgaa |
| si1aF3 | Ggcccttctttaggaaaaatttcaaatctttcaaagctvnnvnnvnnvnnvnnvnnggcctcaataggggtatgcacggttggcccttctttaggaaaaatttcaaatctttcaaagctvnnvnnvnnvnnvnnvnnggcctcaataggggtatgcacggtt |
| si3F3 | ggcccttctttaggaaaaatttcaaatctttcaaagctvnnvnnvnnvnnvnnvnnctccttaattgtttttacatagttgt |
| si4F3 | ggcccttctttaggaaaaatttcaaatctttcaaagctcatttcttactccttaattgtttttaca |
| si8F3 | Ggcccttctttaggaaaaatttcaaatctttcaaagctvnnvnnvnnvnnvnnvnngccatccacgaacacatggtaagttggcccttctttaggaaaaatttcaaatctttcaaagctvnnvnnvnnvnnvnnvnngccatccacgaacacatggtaagtt |
| HAFLAG | tttatcatcgtcatctttataatctaagctaggcccaggatagctcccttctttttcggttaaccataagctaggcccttctttagg  aaaaatttcaaat |
| HAFLAG (rc) | atttgaaatttttcctaaagaagggcctagcttatggttaaccgaaaaagaagggagctatcctgggcctagcttagattataa  agatgacgatgataaa |
| UF1 | Cgactttggttaacccgcaaatcccatcgactttggttaacccgcaaatcccat |
| UR6 | Gggcgtggtggattatgtgtattagggcgtggtggattatgtgtatta |
| si1aR1 | gattataaagatgacgatgataaannbnnbnnbnnbnnbnnbaatggtaaattagttcctggtga |
| si3R1 | tttatcatcgtcatctttataatcvnnvnnvnnvnnvnnctccttaattgtttttacatagttgt |
| si4R1 | gattataaagatgacgatgataaannbnnbnnbnnbnnbnnbaaaaagattagcagaaaagaatatgtt |
| si8R1 | gattataaagatgacgatgataaannbnnbnnbnnbnnbnnbaaagaagtaacttctaaaccagcca |
