# Supplementary material for: Surface Properties of Helicobacter pylori Urease Complex Are Essential for Persistence
Source: PLoS One. 2010 Nov 29;5(11):e15042. doi: 10.1371/journal.pone.0015042 (PMC2993952; doi:10.1371/journal.pone.0015042)
Supplement: Table S2 — Primer combinations used to produce modified DNA. Primers amplified either segments of regions coding exogenous DNA (to be inserted into urease genes) or regions used for homologous recombination after transformation (left flank and right flank). (DOC) [file pone.0015042.s003.doc]

**Table S2.** ***Primer combinations used to produce modified DNA.*** Primers amplified either segments of regions coding exogenous DNA (to be inserted into urease genes) or regions used for homologous recombination after transformation (left flank and right flank).

| Description | Stage | Product | Primer 1 | Primer 2 | Template |
| --- | --- | --- | --- | --- | --- |
| *rpsL,ermB* site 1 |  |  |  |  |  |
|  | 1 | A | UF1 | UR2-234 | genomic |
|  | 1 | B | *rpsL*-F | ermR | genomic^*^ |
|  | 1 | C | UF5-234 | UR6 | genomic |
|  | 2 | Fusion | UF1 | ermR | A+B |
|  | 2 | Fusion | rpsL-F | UR6 | B+C |
|  | 3 | Fusion | UF1 | UR6 | AB+BC |
| *rpsL,ermB* site 8 |  |  |  |  |  |
|  | 1 | A | UF1 | UR2-56 | genomic |
|  | 1 | B | *rpsL*-F | ermR | genomic^*^ |
|  | 1 | C | UF5-56UR6 | UR6 | genomic |
|  | 2 | Fusion | UF1 | ermR | A+B |
|  | 2 | Fusion | rpsL-F | UR6 | B+C |
|  | 3 | Fusion | UF1 | UR6 | AB+BC |
| Site 1 |  |  |  |  |  |
|  | 1 | A1 | UF1 | Si1aR1 | genomic |
|  | 2 | A2 | UF1 | HAFLAG (rc) | A1 |
|  | 1 | B1 | Si1aF3 | UR6 | genomic |
|  | 2 | B2 | HAFLAG | UR6 | B1 |
|  | 3 | Fusion | UF1 | UR6 | A2+B2 |
| Site 3 |  |  |  |  |  |
|  | 1 | A1 | UF1 | Si3R1 | genomic |
|  | 2 | A2 | UF1 | HAFLAG (rc) | A1 |
|  | 1 | B1 | Si3F3 | UR6 | genomic |
|  | 2 | B2 | HAFLAG | UR6 | B1 |
|  | 3 | Fusion | UF1 | UR6 | A2+B2 |
| Site 4 |  |  |  |  |  |
|  | 1 | A1 | UF1 | Si4R1 | genomic |
|  | 2 | A2 | UF1 | HAFLAG (rc) | A1 |
|  | 1 | B1 | Si4F3 | UR6 | genomic |
|  | 2 | B2 | HAFLAG | UR6 | B1 |
|  | 3 | Fusion | UF1 | UR6 | A2+B2 |
| Site 8 |  |  |  |  |  |
|  | 1 | A1 | UF1 | Si8R1 | genomic |
|  | 2 | A2 | UF1 | HAFLAG (rc) | A1 |
|  | 1 | B1 | Si8F3 | UR6 | genomic |
|  | 2 | B2 | HAFLAG | UR6 | B1 |
|  | 3 | Fusion | UF1 | UR6 | A2+B2 |

^*^ Genomic DNA from X47 harbouring *rpsL,ermB* at the *mdaB* locus [1]

1. Dailidiene D, Dailide G, Kersultye D, Berg D (2006) Counterselectable streptomycin susceptibility determinant for genetic manipulation and analysis of Helicobacter pylori. in press.
